# SCN8A Encephalopathy: Case Report and Literature Review

**DOI:** 10.3390/neurolint13020014

**Published:** 2021-04-01

**Authors:** Hueng-Chuen Fan, Hsiu-Fen Lee, Ching-Shiang Chi

**Affiliations:** 1 Department of Pediatrics, Tungs Taichung Metrohabor Hospital, Taichung 435403, Taiwan; fanhuengchuen@yahoo.com.tw; 2 Department of Medical Research, Tungs Taichung Metrohabor Hospital, Taichung 435403, Taiwan; 3 Department of Rehabilitation, Jen-Teh Junior College of Medicine, Nursing and Management, Miaoli 356, Taiwan; 4 Department of Life Sciences, National Chung Hsing University, Taichung 40227, Taiwan; 5 Department of Pediatrics, Taichung Veterans General Hospital, Taichung 40705, Taiwan; leehf@hotmail.com

**Keywords:** SCN8A, SCN8A encephalopathy, oxcarbazepine

## Abstract

Epileptic encephalopathy is a condition resulting from extreme forms of intractable childhood epilepsy. The disease can cause severe delays in cognitive, sensory, and motor function development, in addition to being fatal in some cases. Missense mutations of *SCN8A*, which encodes Nav1.6, one of the main voltage-gated sodium channel subunits in neurons and muscles, have been linked to early infantile SCN8A encephalopathy. Herein, we report the case of a 5-month-old girl with SCN8A encephalopathy with a novel missense mutation. Apart from intractable seizures and autistic phenotypes, the results of blood and biochemical tests, electroencephalogram (EEG) results, and brain magnetic resonance imaging (MRI) results were all normal. As the phenotypes caused by these mutations cannot be identified by any clinical, neuroimaging, or electrophysiological features, genetic sequencing should be considered to identify the underlying genetic causes. Although phenytoin is recommended as a last-resort treatment for SCN8A encephalopathy, the administration of the oxcarbazepine, instead of phenytoin, mitigated this patient’s intractable seizures.

## 1. Introduction

The term “epileptic encephalopathy” (EE) is used to describe conditions in which severe developmental delays, such as delays of cognitive and behavioral development, are mainly caused by unremitting epileptic activity [1]. Recently, the clinical understanding of EE has been altered considerably due to the identification of new genetic variants responsible for numerous severe early onset epilepsies, as well as related changes in public awareness and increasingly early recognition of the associated developmental delays. Therefore, a new term, “developmental epileptic encephalopathy” (DEE), has been introduced for subjects with developmental delays or intellectual impairments resulting from any non-progressive brain condition co-existing along with some form of epilepsy. It should be noted, however, that in some cases, epilepsy itself may directly cause epileptic encephalopathy, whereas in other cases, the onset of the observed development delays may occur before or be totally unrelated to any epileptic seizures or epileptic form abnormalities [2,3]. Accordingly, DEE refers to a range of devastating disorders with heterogeneous etiologies [4,5], and these varying etiologies may in turn lead to delayed or missed diagnosis in some cases.

**Figure 1:**
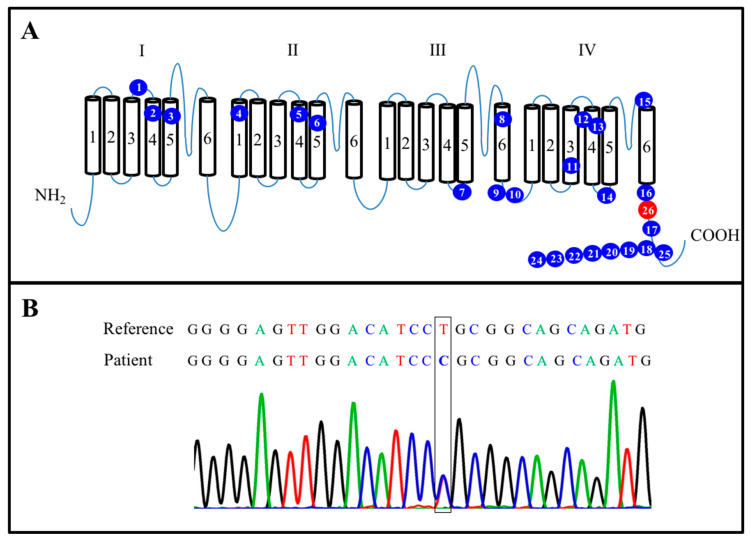
Characterization of mutated SCN8A. (**A**) Summary of 26 novel mutations in the SCN8A protein. The blue spots represent 25 mutated points identified in previously published research. The red spot is located at L1865P (c.5594T > C) position. (**B**) Sanger sequencing confirmed an *SCN8A* (c.5594T > C) mutation in the present case.

Nine genes encoding the voltage-gated sodium channel α subunits Nav1.1–1.9 have been identified and functionally characterized [6]. The *SCN8A* gene encodes the Nav1.6 subunit that forms a complex combined with β subunits, which in turn allows the flow of Na^+^ across cell membranes, maintains electrochemical gradients, and generates action potentials in neurons and muscles [7]. *SCN8A* is composed of four transmembrane domains, each containing six segments (S1–S6), and the four S4 transmembrane segments are responsible for the voltage sensor (Figure 1A) [7,8]. Missense mutations of the *SCN8A* gene have been linked to DEE, and pathogenic variants of *SCN8A* in patients have so far been reported with several phenotypes, including early onset and intractable seizures, intellectual disability, motor disorders, and a relatively high mortality [7,8,9,10]. The mutant channel is seemingly more susceptible to inhibition by phenytoin [11]. In the present article, we present the case of a 5-month-old girl with a novel mutation of *SCN8A* resulting in SCN8A encephalopathy. The administration of the anticonvulsant oxcarbazepine, instead of phenytoin, resulted in the resolution of her intractable seizures. To the best of our knowledge, this case is the first case in Asia and only the second case in the world in which the patient was determined to have the point mutation. The related clinical presentations of this mutation are discussed in detail in the following sections.

## 2. Case Report

The patient was a female infant, G1P1, who was born at a gestational age of 40 3/7 weeks by normal spontaneous delivery with a birth weight of 2900 g and Apgar scores of 8 and 9 at 1 and 5 min, respectively. No family history of seizures was noted. Her body weight was 6.3 kg (15–50th percentile) and her length was 62 cm (15–50th percentile). Her developmental milestones were normal for her age. Seizures were first observed when the child was 5 months old. The frequency of her seizures gradually increased over time from 3 per day to approximately 40 per day. The seizure pattern was characterized by an upward gaze, drooling, neck extension, tonic limbs, and myoclonic seizures of the shoulders, which were accompanied by focal twitching over her left forearm or the absence phenotype. A series of EEG, brain MRI, and biochemical investigations, including a metabolic screening, neurotransmitter studies, and a cerebrospinal fluid work-up, yielded negative results. The seizures persisted despite the administration of phenytoin (loading dose of 20 mg/kg, at least three times on different days, and maintenance dose of 5 mg/kg/day), levetiracetam (30 mg/kg/day), phenobarbiturate (5 mg/kg/day), and valproate (15 mg/kg/day). However, the addition of the oral anticonvulsant oxcarbazepine (4 mg/kg/day) resulted in the resolution of her tonic seizures, and her frequency of seizures was reduced to 0–1 per day when the dose of the oral oxcarbazepine was increased to 20 mg/kg/day and she stopped receiving other AEDs. In addition to seizures, she also presented with an autistic phenotype. A mutation in *SCN8A* (c.5594T > C; p.L1865P) was identified, and the mutation was further confirmed to be a de novo mutation through whole-exome sequencing (Figure 1B). The baby is currently being treated through a rehabilitation program (which includes physical, occupational, and language rehabilitation) to address her developmental delays. She is also visiting a psychiatric clinic for her autism.

## 3. Discussion

Thanks to the increasingly widespread use of genetic screens for patients with epileptic syndrome, more and more *SCN8A*-related phenotypes have been identified in recent years. Pathogenic variants of *SCN8A* were found in 1% of DEEs [9]. The majority of patients with *SCN8A* DEEs have been reported to show early seizure onset, intractable seizures, intellectual disability, motor disorders, and a relatively high mortality [4,12,13,14,15,16,17,18,19,20,21,22,23]. At the same time, *SCN8A* mutations have also been detected in some patients with benign epileptic syndromes and normal intelligence quotient*s* [24]. Patients with ID [25] or movement disorder [26] without epilepsy have also been reported. Given such variations, including wide variations in symptoms and different drug responses among individuals sharing one and the same gene mutation, it is challenging to make early diagnoses and provide proper treatments for patients with *SCN8A* DEEs. In a related effort to obtain a more comprehensive understanding of the clinical presentations of *SCN8A* DEEs and to better predict the altered functions of this mutated point, we searched the PubMed, Google Scholar, and Embase databases using the term“*SCN8A*”and included all related papers that met the following criteria: (1) clinical human studies, (2) reports regarding *SCN8A* variants and/or protein changes, and (3) case reports or original studies. Nine papers meeting these criteria were ultimately selected [12,13,14,15,16,17,18,19,20]. These papers included a total of 26 subjects with mutated *SCN8A* for comparison with the patient presented in the present report. All of the relevant data, except the data for case 26, were adapted from the selected papers and are presented in Table 1.

Analysis of the data showed that there were more male patients than female patients (14 vs.12). The average age of onset among these patients was 3.7 months, with the average age of onset being earlier for the female patients than for the male patients (3.4 ± 4.62 months vs. 4.18 ± 2.04 months).Most of the patients had seizures (88.46%; 23/26), and 80.77% (21/26) of the patients had intractable seizures. Based on data from case reports or series, not from longitudinal studies, only 6 (16.67%) of the patients ultimately became seizure-free, suggesting that most of the cases included in this study consisted of severe *SCN8A* DEEs. Before seizure onset, 80.77% (21/26) of the patients had normal cognitive development. Overall, 96.15% (25/26) of the patients were DD, and 92.31% (24/26) of them were intellectual disability. Fifteen (75%) of the patients were unable to walk autonomously. Although SCN8A is primarily expressed in excitatory neurons with high concentrations at the axon initial segment and the node of Ranvier, where they promote neuronal excitability by participating in the initiation and propagation of action potentials [27,28,29,30,31,32], *SCN8A* seemingly plays an important role in motor function. Studies have shown, in fact, that *SCN8A* is widely expressed in the motor neurons of the brainstem and hippocampus, as well as in the Purkinje and granule cells in the cerebellum [33,34,35]. Therefore, these findings explain why a mutated *SCN8A* gene may result in the manifestation of motor impairments, such as tremor, muscle weakness, ataxia, and dystonia [36,37,38,39,40].

Functionally, *SCN8A* generates persistent current, hyperpolarized thresholds of activation and resurgent current [22]. These electrophysiological propensities make *SCN8A* a critical factor for neuronal firing. Through sequencing, studies of mutated SCN8A channels causing DEEs have made it possible to infer that the pathomechanisms of SCN8A mutations consist of two different types: gain-of-function (GOF) and loss-of-function (LOF) mutations depending on whether the net ionic current flow is Increased or decreased [41]. Certain mutations of *SCN8A*,such as those at the R223G [13], T767I [15], N984K [14], I1327V [16], R1617Q [12,17], N1768D [19], and R1872W [12,20] positions identified in the selected studies, have been suggested to consist of GOF mutations that lead to partial or complete hyperactivity of the sodium channel due to elevated persistent sodium currents, hyperpolarizing shifts in the voltage dependence of activation, or impaired channel current inactivation [18,19,42]. Conversely, other mutations of *SCN8A*, such as those at the R223G and G1451S [12] positions, were found to be associated with partial loss of channel activity, making them LOF mutations. In our case (case 26 in Table 1), the sequencing results showed a missense mutation at the L1865P position, which is located in the C terminal of domain IV of SCN8A (Figure 1A, red spot). To the best of our knowledge, the present case is the first case in Asia and the second case in the world involving a mutation at the L1865P position [43]. The clinical presentations of this mutation are discussed in further detail below.

The mutation at the L1865P position in this case was presumed to be a GOF mutation because of the patient’s good responses to a sodium channel blocker (SCB), oxacarbazepine (OXC; 10,11-dihydro-10-oxo-5Hdibenz[b,f]-azepine-5-carboxamide). OXC, a keto derivative of carbamazepine, has been approved as monotherapy and adjunctive therapy for the treatment of partial seizures with or without secondarily generalized seizures, as well as for the treatment of generalized tonic-clonic seizures [44,45]. OXC inhibits the amplitude of sodium currents in a concentration-dependent manner, produces a negative shift in the steady-state inactivation curve of sodium currents, prolongs the recovery of sodium current inactivation, and decreases the conductance of sodium currents, leading to a lessening of the excitability of neurons that in turn prevents the over-excitation that leads to seizures [46]. Phenytoin (5,5-diphenyl-imidazolidine-2,4-dione), meanwhile, binds at a receptor site in the pore of sodium channels and decreases sodium influx, thereby decreasing the excitability of neurons and preventing the further generation of seizures [47]. Moreover, phenytoin decreases calcium influx into neurons to abolish the release of neurotransmitters [48], modulates GABA and glutamate release [49], and reduces synaptic post-tetanic potentiation and excitatory synaptic transmission to stop the abnormal cortical current propagation [50]. At the same time, phenytoin significantly prevents generalized tonic-clonic seizures, complex partial seizures, and status epilepticus through those mechanisms. Moreover, it has been suggested that phenytoin only targets the mutated Nav1.6 subunit while not affecting the functions of any other sodium channels [14], and therefore it has been reported to yield remarkably good seizure control in several cases resulting from *SCN8A* missense mutations, such that it can serve as a last-resort treatment for SCN8A encephalopathy [11]. Table 1 shows that 100% (21/21) of the patients included in this study for whom AED data were available were, in fact, exposed to AEDs. Further analysis indicated that 52.38% (11/21) of those patients received phenytoin and had intractable seizures, while only two of the individuals were seizure-free. Of the patients who did not receive phenytoin, 90% had intractable seizures and only one individual was seizure-free, suggesting that patients with mutated *SCN8A*, whether treated with or without phenytoin, mostly had intractable conditions with poor prognosis.

Sequencing has revolutionized the detection of disease-causing mutations, with such detection being of value not only for research purposes, but also for the diagnosis and treatment of affected patients. As different *SCN8A* mutations can have a variety of different effects, for example, with different R223G mutations being both GOF [13] and LOF [13] mutations, these differences may explain why SCBs, such as oxacarbazepine and phenytoin, with similar mechanisms are different with respect to their effects in treating *SCN8A*-related epileptic encephalopathy. Dravet syndrome, another mutated sodium channel disease, substantially reduces whole-cell sodium currents and action potentials in inhibitory interneurons, leading to increased action-potential generation and firing frequency [51]. Relatedly, patients with this disease may deteriorate if treated with phenytoin and oxcarbazepine. Through sequencing, clinicians, patients, and medical caregivers can clearly understand the identified mutations in affected children that result in missense substitutions of evolutionarily conserved amino acid residues that in turn alter channel activity, thereby allowing them to specifically differentiate those similar sodium channel diseases and precisely administer specific medications to the affected patients. Moreover, knowing an individual variant’s function is crucial because it may provide further detailed information on clinical manifestations (whether present or not), predictive therapeutic responses, and prognosis, and prevent the administration of incorrect treatments. Although experimental studies of channel function are also very important, laboratory tests to clarify the functions of variants are expensive, time-consuming, focus on only a few targets, and require relevant expertise. Moreover, manual analysis of the mounting data is not practical. Sophisticated statistical and computational tools (in silico) have been developed to analyze large quantities of data and to study the ion channel structure-function relationship [52]. Recently, a new technique using a machine learning statistical model was developed to predict LOF or GOF pathogenic variants’ effects, thus providing valuable information regarding the clinical genetics and functional variants of genes of channels to patients, families, and clinicians [53]. This program has the potential to be adapted and benchmarked for use in conjunction with best current clinical practices if the program can be integrated with more DEE patients’ clinical data and refined with large-scale experimental data.

A mutated *SCN8A* gene can lead to a variety of unique clinical presentations, such as focal and/or generalized seizures (tonic, myoclonic, absence), epileptic spasms with a normal EEG background activity, variable psychomotor delays after seizure onset, normal brain MRI results, and rare febrile seizures [8,54,55,56]. As such, for cases of epileptic encephalopathy in which the patient exhibits normal blood and biochemical investigation results, normal brain MRI and EEG results, and no reduction in seizures after pyridoxine or pyridoxal-5-phosphate treatment, it is recommended that targeted sequencing be applied to identify the underlying genetic causes. Also, these substantial functional differences in the effects of *SCN8A* mutations further bolster the case for using gene sequencing to identify the specific mutations underlying cases of epileptic encephalopathy. Such an approach may provide researchers with novel insights into the pathogenesis of the given disease, inform prognostic counseling, guide clinicians in choosing appropriate treatments, and aid in the development of targeted neuroprotective treatment strategies that could substantially enhance the long-term health outcomes of epileptic encephalopathy patients. Importantly, the patient in this case demonstrated a favorable response to oxcarbazepine.

## 4. Conclusions

The clues indicating DEE with *SCN8A* mutation are best discovered through a careful history taking, thorough physical examination, laboratory tests, brain imaging, and in particular, a high level of suspicion. The case described in the present report has brought to our attention the fact that when a patient with epileptic encephalopathy exhibits normal blood and biochemical investigation results, normal brain MRI and EEG results, and no reduction in seizures after pyridoxine or pyridoxal-5-phosphate treatment, targeted sequencing may provide useful information to uncover the underlying genetic causes. Although SCBs such as phenytoin are recommended as a last-resort treatment for SCN8A encephalopathy, the administration of oxcarbazepine may be considered in mutated *SCN8A* patients with intractable seizures, as that treatment appeared to be helpful at least in this case.

## Figures and Tables

**Table 1:** Summary of 26 patients carrying an SCN8A mutation.

Case	Position	PM	Gender	Onset Age	SZ	IT	SF	DD	ID	WA	AEDs	PHT	Ref
1	V216D	GOF	F	7 mo	+	+	−	+	+	−	+	NE	[12]
2	R223G	GOF/LOF	F	6 mo	+	+	−	+	+	−	+	NE	[13]
3	F260S	NA	F	4 mo	+	+	−	+	+	−	+	E	[14]
4	T767I	GOF	M	0	+	+	−	+	+	NA	+	NE	[15]
5	F864S	NA	M	0	+	+		+	+	−	+	E	[12]
6	N984K	GOF	M	6 wk	+	+	+	+	+	−	+	E	[14]
7	I1327V	GOF	M	0	+	+	−	+	+	−	+	E	[16]
8	G1451S	LOF	M	18 mo	−	−	−	+	+	+	NA	NA	[12]
9	N1466K	NA	M	3 d	+	+	−	+	+	−	+	E	[12]
10	N1466T	NA	M	4 mo	+	+	−	+	+	+	+	E	[12]
11	S1596C	NA	M	5 mo	+	+	+	+	+	NA	+	E	[14]
12	R1617Q	GOF	F	3 mo	+	+	−	+	+	−	+	NE	[12]
13	R1617Q	GOF	F	NA	NA	NA	NA	+	+	NA	NA	NA	[17]
14	A1650T	NA	M	3.5 mo	+	+	−	+	+	−	+	NE	[12]
15	P1719R	NA	M	0	−	−	−	+	+	−	NA	NA	[18]
16	N1768D	GOF	F	0	+	+	NA	+	+	+	NA	NA	[19]
17	R1872W	GOF	F	3 mo	+	+	+	+	+	−	+	NE	[14]
18	R1872W	GOF	F	4 mo	+	+	−	+	+	−	+	NE	[12]
19	R1872W	GOF	F	7 mo	+	+	−	+	+	+	NA	NA	[20]
20	R1872W	GOF	F	4 mo	+	+	−	+	+	NA	+	E	[20]
21	R1872W	GOF	M	4 mo	+	+	−	+	+	NA	+	E	[20]
22	R1872W	GOF	M	4 mo	+	+	−	+	+	+	+	NE	[20]
23	R1872W	GOF	F	3 mo	+	+	−	+	+	−	+	E	[20]
24	R1872W	GOF	M	4 mo	+	−	−	−	−	−	+	NE	[20]
25	E1870D	NA	M	3.5 mo	+	+	−	+	+	NA	+	E	[20]
26	L1865P	NA	F	5 mo	+	+	+	+	+	−	+	E	Fan et al.

PM: predictive mechanism; GOF: gain-of-function; LOF: loss-of-function; SZ: seizures; IT: intractable seizures; SF: seizure free; DD: developmental delay; ID: intellectual disability; WA: walk autonomously; AEDs: anti-epileptic drugs; PHT: phenytoin; NA: not available; E: expose; NE: not expose.

## Data Availability

All data related to this case report are contained within the manuscript.
